# The role of blood CXCL12 level in prognosis of coronary artery disease: A meta-analysis

**DOI:** 10.3389/fcvm.2022.938540

**Published:** 2022-07-27

**Authors:** Shunrong Zhang, Yu Ding, Fei Feng, Yue Gao

**Affiliations:** ^1^Department of Geriatrics, Affiliated Hangzhou First People's Hospital, Zhejiang University School of Medicine, Hangzhou, China; ^2^Central Laboratory, Affiliated Hangzhou First People's Hospital, Zhejiang University School of Medicine, Hangzhou, China

**Keywords:** CXCL12, coronary artery disease, acute coronary syndrome, prognosis, MACEs, meta-analysis

## Abstract

**Objective:**

The role of C-X-C motif chemokine 12 (CXCL12) in atherosclerotic cardiovascular diseases (ASCVDs) has emerged as one of the research hotspots in recent years. Studies reported that the higher blood CXCL12 level was associated with increased major adverse cardiovascular events (MACEs), but the results were inconsistent. The objective of this study was to clarify the prognostic value of the blood CXCL12 level in patients with coronary artery disease (CAD) through meta-analysis.

**Methods:**

All related studies about the association between the blood CXCL12 level and the prognosis of CAD were comprehensively searched and screened according to inclusion criteria and exclusion criteria. The quality of the included literature was evaluated using the Newcastle-Ottawa Scale (NOS). The heterogeneity test was conducted, and the pooled hazard risk (HR) or the odds ratio (OR) with a 95% confidence interval (CI) was calculated using the fixed-effect or random-effects model accordingly. Publication bias was evaluated using Begg's funnel plot and Egger's test. Sensitivity analysis and subgroup analysis were also conducted.

**Results:**

A total of 12 original studies with 2,959 CAD subjects were included in the final data combination. The pooled data indicated a significant association between higher CXCL12 levels and MACEs both in univariate analysis (HR 5.23, 95% CI 2.48–11.04) and multivariate analysis (HR 2.53, 95% CI 2.03–3.16) in the CXCL12 level as the category variable group. In the CXCL12 level as the continuous variable group, the result also indicated that the higher CXCL12 level significantly predicted future MACEs (multivariate OR 1.55, 95% CI 1.02–2.35). Subgroup analysis of the CXCL12 level as the category variable group found significant associations in all acute coronary syndrome (ACS) (univariate HR 9.72, 95% CI 4.69–20.15; multivariate HR 2.47, 95% CI 1.79–3.40), non-ACS (univariate HR 2.73, 95% CI 1.65–4.54; multivariate HR 3.49, 95% CI 1.66–7.33), Asian (univariate HR 7.43, 95% CI 1.70–32.49; multivariate HR 2.21, 95% CI 1.71–2.85), Caucasian (univariate HR 3.90, 95% CI 2.73–5.57; multivariate HR 3.87, 95% CI 2.48–6.04), short-term (univariate HR 9.36, 95% CI 4.10–21.37; multivariate HR 2.72, 95% CI 1.97–3.76), and long-term (univariate HR 2.86, 95% CI 1.62–5.04; multivariate HR 2.38, 95% CI 1.76–3.22) subgroups. Subgroup analysis of the CXCL12 level as the continuous variable group found significant associations in non-ACS (multivariate OR 1.53, 95% CI 1.23–1.92), Caucasian (multivariate OR 3.83, 95% CI 1.44–10.19), and long-term (multivariate OR 1.62, 95% CI 1.37–1.93) subgroups, but not in ACS (multivariate OR 1.36, 95% CI 0.67–2.75), Asian (multivariate OR 1.40, 95% CI 0.91–2.14), and short-term (multivariate OR 1.16, 95% CI 0.28–4.76) subgroups. No significant publication bias was found in this meta-analysis.

**Conclusion:**

The higher blood CXCL12 level is associated with increased MACEs in patients with CAD, and the blood CXCL12 level may serve as an important prognostic index for CAD. Integrating the blood CXCL12 level into CAD risk assessment tools may provide more comprehensive messages for evaluating and managing patients with CAD.

## Introduction

Heart disease is the leading cause of death worldwide. As the most common type of heart disease, coronary artery disease (CAD) also referred to as coronary heart disease (CHD) or ischemic heart disease (IHD) affects around 126 million individuals globally, which is estimated to be 1.72% of the world's population in 2017 (1). In China, with the aging of the population, the prevalence and mortality of CAD have been increasing continuously within the past two decades (2). Although with the progress of medical care, the prognosis of CAD is still not optimistic, especially in acute coronary syndrome (ACS) (3) and elderly patients (4). Thus, in addition to diagnosis and therapy, evaluation of prognosis or risk stratification for patients with CAD is a clinical matter of great concern.

In fact, many risk stratification tools, such as the GRACE and CRUSADE scores for assessing the risk of patients with non-STEMI ACS (5), have been generated for risk classification for CAD. Although each may have its respective merits, these risk stratification tools are not comprehensive or have some limitations. Thus, exploring new strategies or indicators guiding more precise evaluation of CAD prognosis and directing more optimized treatment of CAD is of great clinical significance. In recent years, the clinical prognostic value of novel biomarkers in CAD has increasingly aroused people's attention (6).

The C-X-C motif chemokine 12 (CXCL12), also known as stromal cell-derived factor-1 (SDF-1), is a chemokine protein that exerts multifaceted roles in atherosclerosis and other cardiovascular diseases through its classical C-X-C motif chemokine receptor 4(CXCR4) and atypical ACKR3 (atypical chemokine receptor 3, also CXCR7) receptors (7, 8). The role of the CXCL12/CXCR4/ACKR3 system in the pathogenesis of cardiovascular diseases was a research hotspot in recent years. Studies reported that CXCL12 gene polymorphisms are associated with an increased risk of CAD (9, 10), and a high blood CXCL12 level predicted high coronary events in diabetes patients (10).

Other studies reported that an increased level of blood CXCL12 predicted adverse clinical outcomes in CAD. Chang et al. first reported that a higher serum CXCL12 level positively predicted 30-day major adverse clinical outcomes in patients with acute myocardial infarction (AMI) (11). Thereafter, several studies supported the positive correlation between higher blood CXCL12 levels and increased risk of future (both short and long terms) adverse clinical outcomes in patients with CAD (12–14). However, negative or even opposite results were also reported, which found no significant correlation between blood CXCL12 levels and CAD prognosis (15), or even higher serum CXCL12 levels predicting lower future cardiovascular events in patients with CAD (16). Thus, the association between blood CXCL12 levels and future major adverse cardiovascular events (MACEs) in patients with CAD seems to be controversial.

A good method to resolve the contradictions between individual studies is meta-analysis. To evaluate the predicting role of the blood CXCL12 level in the prognosis of CAD objectively, we reviewed all the related literature comprehensively and conducted a meta-analysis.

## Methods

### Search strategy

All related studies about the correlation between blood CXCL12 level and CAD prognosis were identified by comprehensive computer-based searches. The retrieved databases included PubMed, EMBASE, ScienceDirect, Web of Science, and the China National Knowledge Infrastructure (CNKI) database. The keywords used for the literature search were combined as follows: (“CXCL12” OR “C-X-C motif chemokine ligand 12” OR “SDF-1” OR “stromal cell-derived factor-1 ”) and (“coronary artery disease” OR “coronary heart disease” OR “CAD” OR “CHD” OR “ischemic heart disease” OR “myocardial infarction” OR “angina” OR “acute coronary syndrome” OR “STEMI” OR “non-STEMI”) and (“prognosis” OR “MACE” OR “major adverse cardiovascular events” OR “adverse outcome”). The last search was updated on 8 June 2022, and the literature language was limited to English and Chinese.

### Data inclusion and exclusion criteria

#### Data inclusion criteria

The inclusion criteria for eligible studies were as follows: (1) studies evaluated the association between the blood CXCL12 level and the prognosis of CAD. (2) The CAD diagnostic criteria were angiographically confirmed CAD or ACS diagnosed with general standard criteria. (3) Studies were published in prospective cohort studies. (4) The follow-up duration was at least 30 days. (5) The actual number of MACEs was presented, or the hazard ratio (HR)/odds ratio (OR) and 95% confidence interval (CI) of blood CXCL12 level and MACEs were provided.

#### Data exclusion criteria

Exclusion criteria included (1) conference abstracts or reviews; (2) unpublished data; (3) studies with duplicated publications or studies with partially replicated populations; (4) primary endpoints and secondary endpoints were not about death or adverse cardiovascular events; and (5) Newcastle-Ottawa Scale (NOS) score was <6 scores.

### Data extraction

Three reviewers (Zhang, Ding, and Feng) extracted key data from each included original study independently, and the extracted data included the name of the first author, publication year, study type, sample size, region, ethnicity of the study population, diagnostic criteria for patients with CAD, methods for measuring CXCL12 level, cutoff or comparison of CXCL12 level, follow-up duration, measurement of clinical outcomes, and covariables adjusted in the multivariate model. For studies in which the CXCL12 level was presented as a continuous variable, we standardized the group-level exposure estimates to single units, thereby allowing for combining the effects of different CXCL12 values in different studies. All the independently extracted data were compared, and disagreements were settled by consensus. If these three authors could not reach a consensus, the results were further arbitrated by the fourth author (Gao).

### Literature quality assessment

The quality was assessed and scored using the Newcastle-Ottawa Quality Assessment Scale (NOS) (17) system by two authors independently. The NOS uses a “star” rating system ranging from zero (worst) to nine stars (best) to judge the quality of observational studies, and studies with a total score of ≥7 were generally regarded as high quality. Any disagreements about study quality assessment between the two authors were settled by consensus or consulted by the third author.

### Statistical analysis

STATA 16.0 (STATA Corp., College Station, TX, USA) was used to carry on the statistical analysis. The pooled HRs or ORs and 95% CIs were used as the effect indicator to evaluate the predicting role of the blood CXCL12 level in CAD prognosis. According to the different variable types of the CXCL12 level used in each original study, all the included studies were divided into the CXCL12 level as the category variable group and the CXCL12 level as the continuous variable group, and the overall effects were combined separately. Heterogeneity between studies was assessed using the *I*^2^ test, and *I*^2^ > 50% and *P* < 0.1 were considered existing significant heterogeneity (18). If significant heterogeneity was found, the random-effects pooling model (I-V heterogeneity) was used to evaluate the pooled HRs or ORs (with 95% CIs); otherwise, the fixed-effect pooling model (inverse variance) was used to calculate pooled HRs or ORs (with 95% CIs). The significance of overall effects was tested using the *Z*-test (19). Subgroup analysis was performed based on different ethnicities, CAD types, and follow-up durations to explore the predicting role of the blood CXCL12 level in the CAD prognosis more comprehensively. Sensitivity analysis was conducted to observe the influence of any single study on the pooled HRs or ORs to evaluate the robustness of overall effects. The potential publication bias was assessed using Begg's funnel plot and Egger's test. Except for the *I*^2^ test for assessing heterogeneity, a 2-tailed *P* < 0.05 was considered to be statistically significant.

## Results

### Literature search and study characteristics

**Figure 1:**
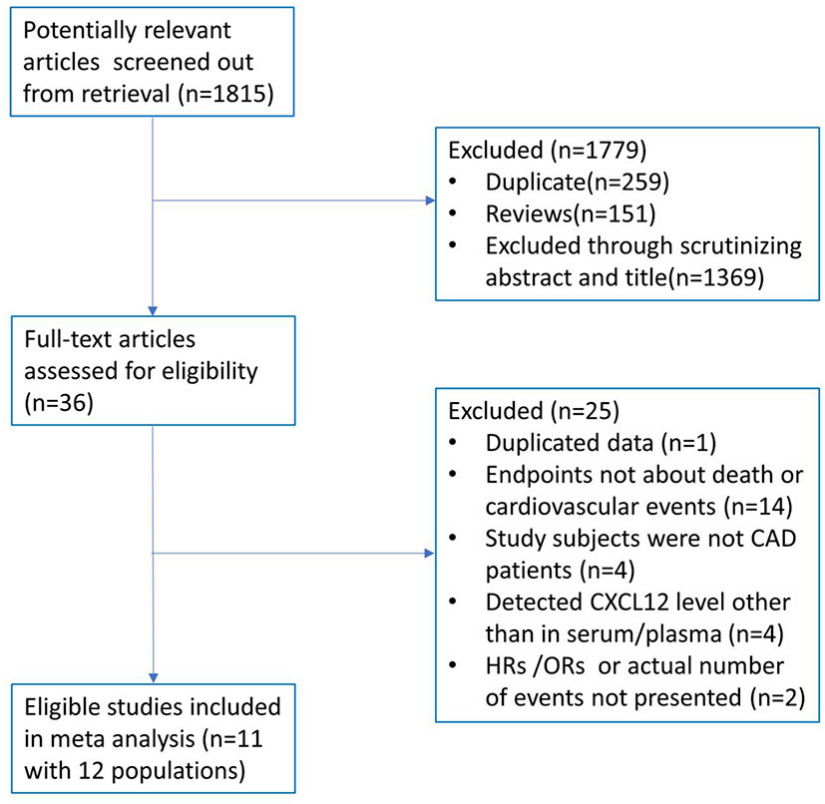
Flow diagram of the study selection process.

A total of 1,815 potentially relevant articles were initially identified according to the search criteria described above. After screening titles and abstracts, 1,779 studies were excluded for duplicates, reviews, or being irrelevant. The left 36 articles were entered full-text assessment for eligibility, and 25 articles were further excluded for duplicate data, endpoints not about death or cardiovascular events, study subjects were not patients with CAD, detected CXCL12 level not in serum/plasma, or could not get outcome measurement data. As a result, a total of 11 articles (12 studies) with 2,959 patients with CAD were included in this meta-analysis for the final data combination. The study screening process is shown in Figure 1.

Of the included 12 studies, four studies enrolled patients with STEMI as study subjects, four studies enrolled patients with CAD as study subjects, and the left four studies enrolled patients with the acute coronary syndrome. As for the methods for measuring the CXCL12 level, all the included original studies detected the CXCL12 level by enzyme-linked immunosorbent assay (ELISA), with seven studies detected in serum and the left 5 studies in plasma. According to the variable type of the CXCL12 level adopted by the authors, the included studies were divided into two groups: one group incorporated 6 studies with the CXCL12 level as the category variable, and the other group consisted of the left sixstudies with the CXCL12 level as the continuous variable. The pooled data of the two groups were calculated separately. The main characteristics of the included studies are presented in Table 1.

**Table 1:** Clinical characteristics of the included original studies.

**Study**	**Region**	**Subjects**	**Study design**	**Sample size**	**Follow up (month)**	**Methods for measuring SDF-1 level**	**CXCL12 Cutoff or comparison (pg/ml)**	**Outcome endpoints**	**HR/OR (95%CI)**		**Adjusted covariates in multivariate analysis**	**NOS score**
									**Univariate**	**Multivariate**		
Matsuoka et al. (20)	Japan	OMI	Prospective study	192	90	Detected with ELISA in plasma	≥2,162 vs. <2,162	Cardiac death, non-fatal MI, refractory unstable angina pectoris (UAP), decompensated heart failure	1.87 (1.35–2.60)	1.98 (1.38–2.85)	Age, gender, smoking, hypertension, diabetes, multivessel disease, BMI, heart rate, LDL, HDL, HbA1c, LVEF, GFR, BNP, CRP, aspirin, thienopyridines, b-Blocker, ACEI/ARB, statin	7
Ghasemzadeh et al. (12)	USA	CAD	Prospective study	186	67.7	Detected with ELISA in plasma	>1,734 vs. <1,734	CV death, MI	3.01 (1.68–5.40)	6.24 (2.61–14.91)	Age, gender, diabetes, hypertension, smoking, acute MI, serum creatinine, LVEF, history of CABG, statin use, aspirin use, presence of at least 50% stenosis in at least one major epicardial vessel, LDL	7
Ghasemzadeh et al. (12)	USA	CAD	Prospective study	599	19.2	Detected with ELISA in plasma	>2,679 vs. <2,679	CV death, MI	4.27 (2.30–7.91)	4.36 (2.05–9.28)	Age, gender, diabetes, hypertension, smoking, acute MI, serum creatinine, LVEF, history of CABG, statin use, aspirin use, presence of at least 50% stenosis in at least one major epicardial vessel, LDL	7
Tong et al. (13)	China	ACS	Prospective study	678	18	Detected with ELISA in plasma	>2175.1 vs. <2175.1	Death, recurrent MI, advanced HF	10.879 (7.635–15.499)	2.45 (1.71–3.50)	Age, gender, BMI, smoking, diabetes, hypercholesterolemia, hypertension, MI, chronic HF, revascularization, ST-depression ≥0.1 mV, troponin I, GFR, delay time, admission to balloon time, Killip class, left main artery disease, triple vessel disease, NT-proBNP, hs-CRP, ACEI, ARB, β-blocker, statin, aspirin, clopidogrel, tirofiban	7
Chang et al. (11)	China (Taiwan)	AMI	Prospective study	129	1	Detected with ELISA in serum	>1,500 vs. ≤ 1,500	Advanced Killip score, mortality	26.00 (7.20–93.93)	NA	NA	7
Peir' (14)	Spain	ACS	Prospective study	254	60	Detected with ELISA in plasma	Three tertile vs. 1+2 tertile	All-cause death	4.90 (2.53–9.50)	2.53 (1.24–5.16)	Age, medical history of myocardial infarction, diabetes, chronic kidney disease, GRACE score, troponin I peak, three vessels stenosis, LVEF ≤ 40%, NSTEMI or unstable angina	7
Cai et al. (21)	China	CAD	Prospective study	130	1	Detected with ELISA in serum	Continuous variable	CV death, recurrent MI, advanced HF	NA	3.683 (1.131–11.989)	White blood cell, mean platelet volume, erythrocyte mean volume, hs-CRP, CTnI, BNP, LVEF, apolipoprotein A	6
										apolipoprotein B, lipoprotein a, TG, TC, HDL, LDL	
Zhang et al. (22)	China	ACS	Prospective study	214	6	Detected with ELISA in serum	Continuous variable	Total death, CV death, recurrent MI, recurrent angina, stroke, advanced HF	NA	1.812 (1.187–2.767)	Age, gender, BMI, smoking, diabetes, hypertension, hyperlipidemia, CAD family history, LVEF, hs-CRP	6
Yang et al. (23)	China	CHD	Prospective study	189	12	Detected with ELISA in serum	Continuous variable	CV death, recurrent MI, revascularization, in-stent thrombosis	NA	1.484 (1.183–1.863)	Hypertension, hyperlipidemia, diabetes, number of coronary artery lesions, length of coronary artery lesions, Gensini scores, LVEF, CRP, TNF-α	6
Yang et al. (24)	China	AMI	Prospective study	94	12	Detected with ELISA in serum	Continuous variable	CV death, recurrent angina, advanced HF, malignant ventricular arrhythmia	NA	1.733 (1.317–2.281)	Age, gender, BMI, smoking, alcohol consumption, diabetes, blood pressure, TC, TG, HDL, LDL	6
Cai et al. (16)	China	STEMI	Prospective study	122	10	Detected with ELISA in serum	Continuous variable	CV death, recurrent MI, recurrent angina, advanced HF, malignant ventricular arrhythmia	NA	0.246(0.1–0.603)	Age, hypertension, diabetes, TC, TG, HDL, LDL, creatinine level, cTnI, white blood cell, fast glucose level	6
Fortunato et al. (25)	Italy	AMI	Prospective study	172	12	Detected with ELISA in serum	For 1,000 U increase	Death, repeat AMI, new-onset heart failure	3.4(1.6–9.99)	3.83(1.44–10.19)	Age, gender, the presence of ST elevation, and diabetes	6

*ACEI, angiotensin-converting enzyme inhibitor; AMI, acute myocardial infarction; ARB, angiotensin receptor blocker; BMI, body mass index; BNP, B type natriuretic peptide; CABG, coronary artery bypass grafting; CAD, coronary artery disease; CHD, coronary heart disease; CRP, C-reactive protein; cTnI, cardiac troponin I; CV, cardiovascular; ELISA, enzyme-linked immunosorbent assay; GFR, glomerular filtration rate; HbA1c, glycosylated hemoglobin A1c; HDL, high-density lipoprotein; HF, heart failure; hs-CRP, hypersensitive C-reactive protein; LDL, low-density lipoprotein; LVEF, left ventricular ejection fraction; MI, myocardial infarction; NA, not available; NOS, Newcastle-Ottawa Scale; NT-proBNP, N-terminal pro-B type natriuretic peptide; OMI, old myocardial infarction; TC, total cholesterol; TG, triglyceride; STEMI, ST-segment elevation myocardial infarction; TNF-α, tumor necrosis factor-α; UAP, unstable angina pectoris*.

### CXCL12 level and future clinical outcomes in patients with CAD

**Figure 2:**
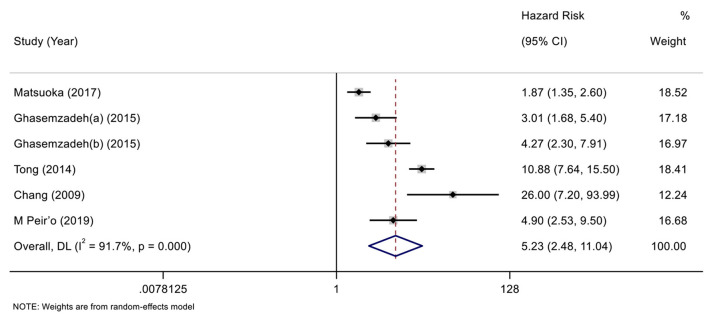
Forest plot of the association between C-X-C motif chemokine 12 (CXCL12) level as category variable and major adverse cardiovascular events (MACEs) in patients with coronary artery disease (CAD) (univariate analysis).

**Figure 3:**
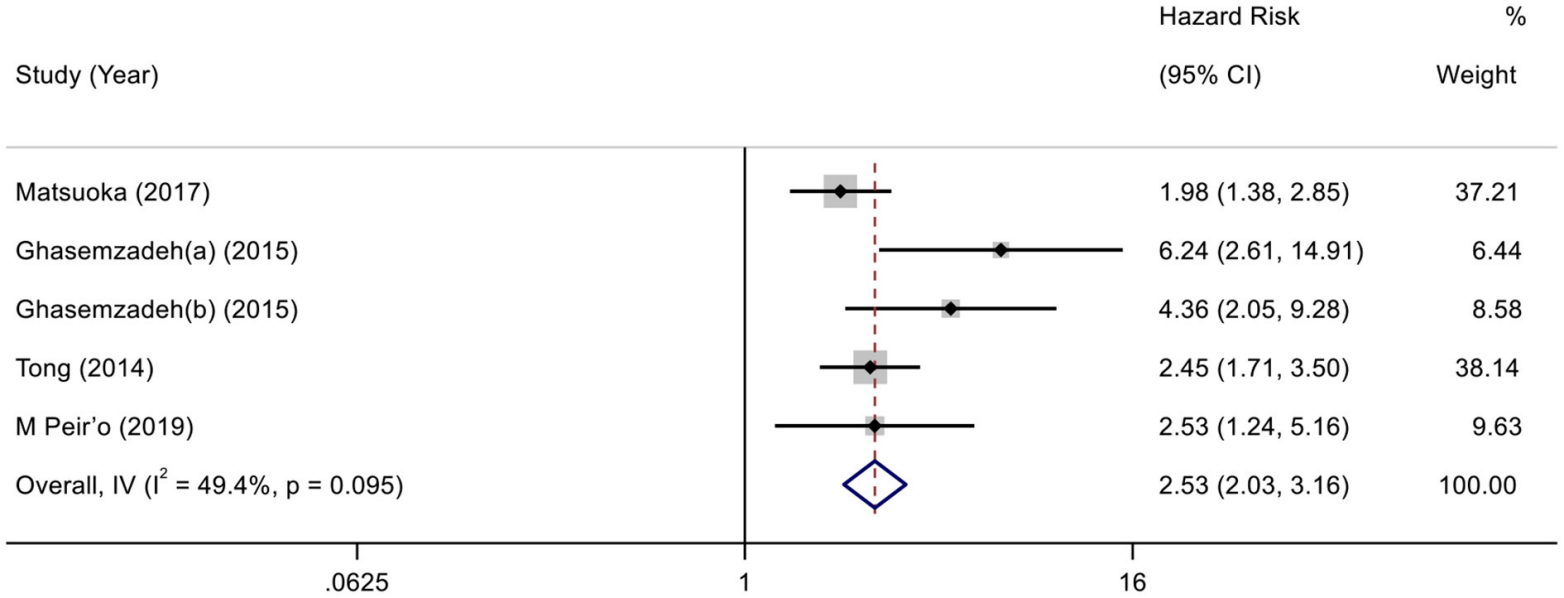
Forest plot of the association between CXCL12 level as category variable and MACEs in patients with CAD (multivariate analysis).

**Figure 4:**
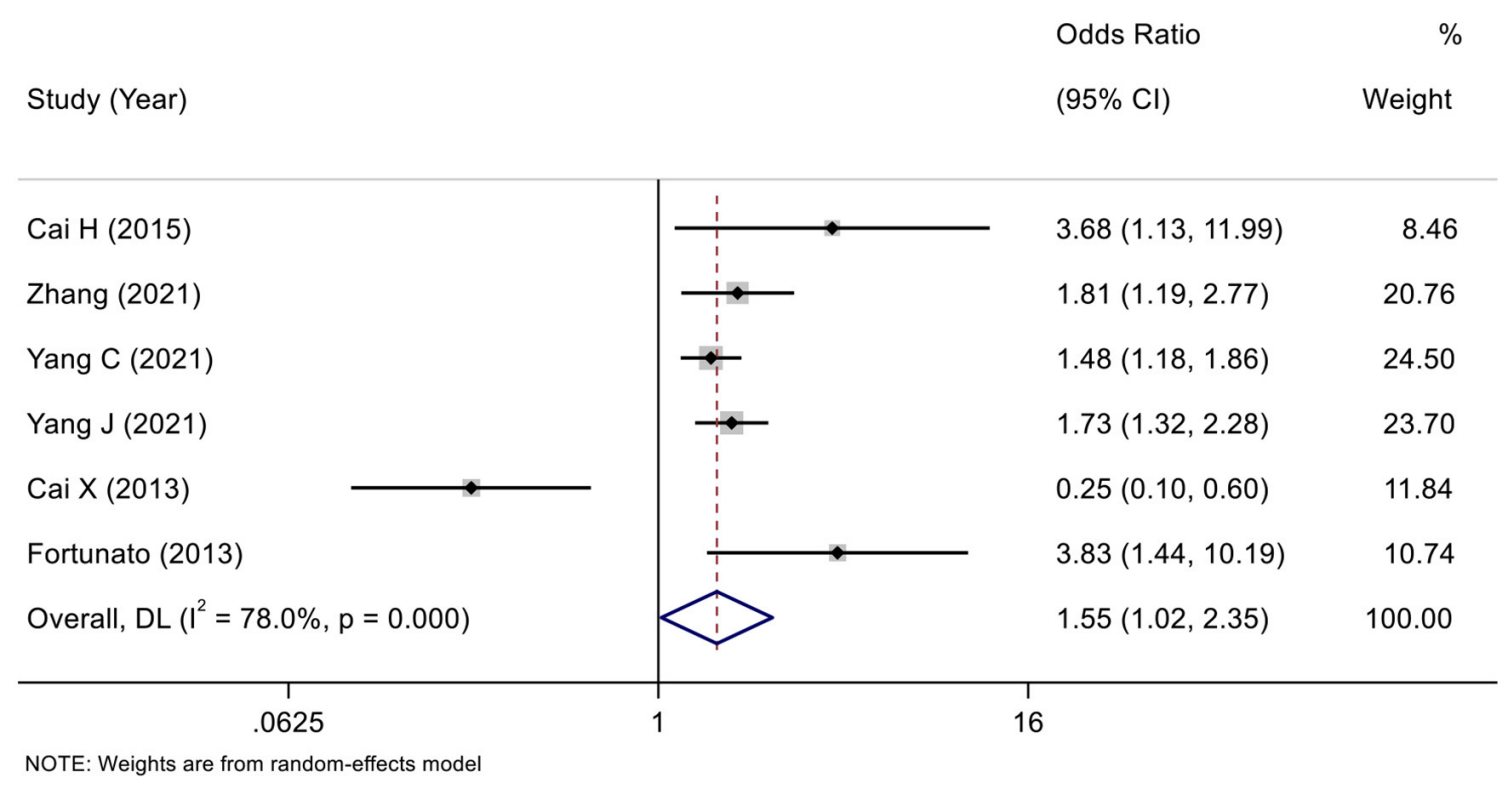
Forest plot of the association between CXCL12 level as continuous variable and MACEs in patients with CAD (multivariate analysis).

According to the variable type of CXCL12 level adopted in the original studies, all the included original studies were divided into CXCL12 level as the category variable group and CXCL12 level as the continuous variable group, and we pooled the data of the two groups separately. Before calculating pooled HRs/ORs, a heterogeneity test was conducted. In CXCL12 level as category group, heterogeneity was found in univariate analysis (*I*^2^ = 91.7%, *P* < 0.001) but not in multivariate analysis (*I*^2^ = 49.4%, *P* = 0.095) (Figures 2, 3). In the CXCL12 level as the continuous variable group, heterogeneity was found in multivariate analysis (*I*^2^ = 78%, *P* < 0.001) (Figure 4), while univariate analysis data could not be pooled for only one study presented univariate OR and 95% CI. Thus, a random-effects model was used to merge HRs/ORs in univariate analysis of CXCL12 level as category variable group and in CXCL12 level as the continuous variable group, while a fixed-effect model was used to merge HRs in multivariate analysis of CXCL12 level as the category group. A positive association between higher blood CXCL12 level and future MACEs was found in both CXCL12 level as category variable group (univariate HR 5.23, 95% CI 2.48–11.04, *P* < 0.001; multivariate HR 2.53, 95% CI 2.03–3.16, *P* < 0.001) (Figures 2, 3) and CXCL12 level as continuous variable group (multivariate OR 1.55, 95%CI 1.02–2.35, *P* = 0.039) (Figure 4).

### Publication bias

**Figure 5:**
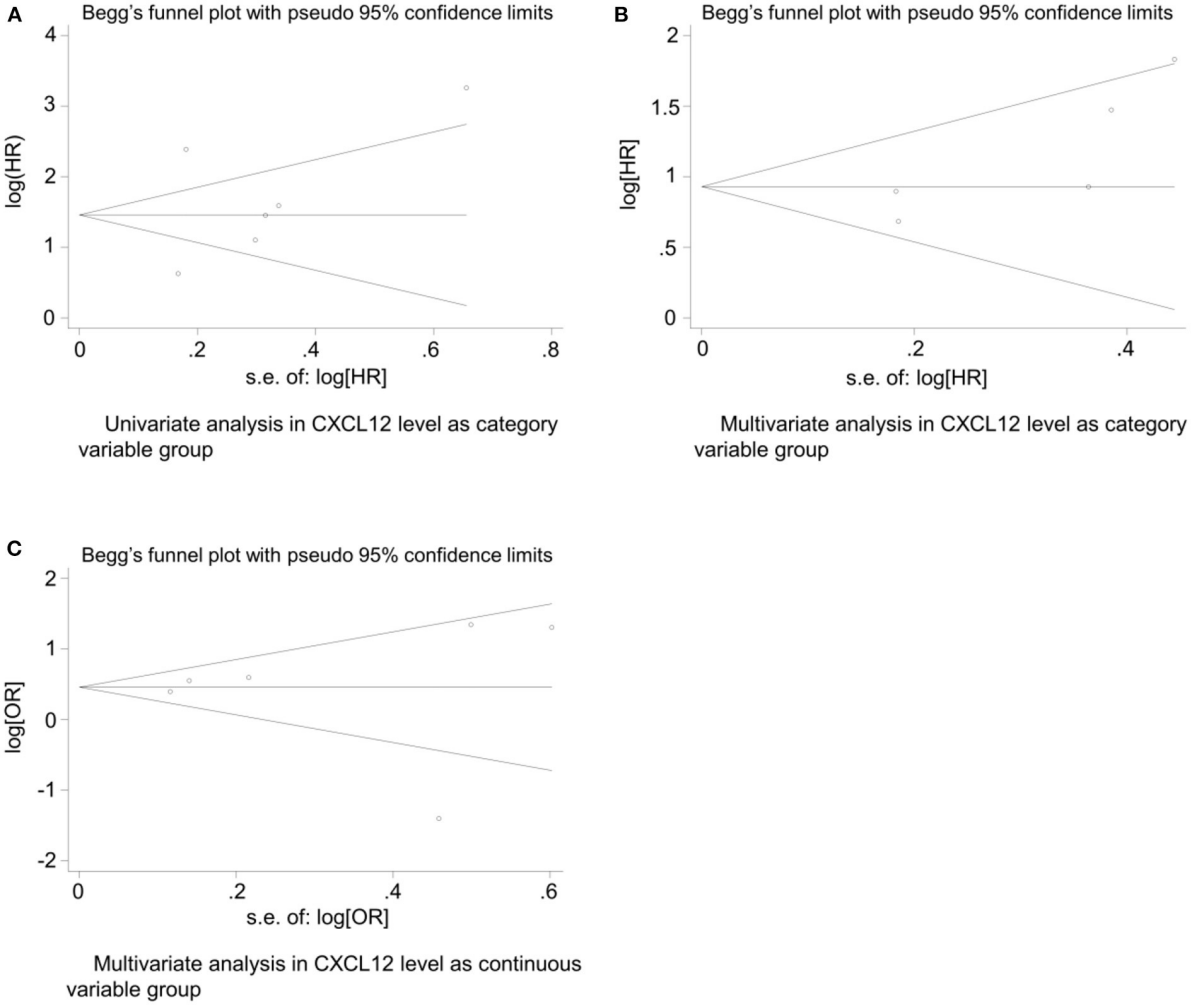
Begg's funnel plot for evaluating publication bias of included studies. **(A)** Univariate analysis in CXCL12 level as category variable group (*Z* = 1.13, *P* = 0.26); **(B)** multivariate analysis in CXCL12 level as category variable group (*Z* = 1.71, *P* = 0.086); **(C)** and multivariate analysis in CXCL12 level as continuous variable group (*Z* = 0.75, *P* = 0.452).

The Egger's (26) test and Begg's funnel plot were used to evaluate the publication bias of the included studies in both the CXCL12 level as the category group and the CXCL12 level as the continuous group. Begg's test found no significant publication in all the univariate analysis of CXCL12 level as category group (*Z* = 1.13, *P* = 0.26), the multivariate analysis of CXCL12 level as category group (*Z* = 1.71, *P* = 0.086), and the multivariate analysis of CXCL12 level as continuous group (*Z* = 0.75, *P* = 0.452). No obvious asymmetry was found in Begg's funnel plots for all these three analyses (Figure 5). Since Egger's test has a higher sensitivity than Begg's test, Egger's test was further conducted. In addition, Egger's test also found no significant publication in all the univariate analysis of CXCL12 level as category group (*t* = 0.61, *P* = 0.577), the multivariate analysis of CXCL12 level as category group (*t* = 2.71, *P* = 0.073), and the multivariate analysis of CXCL12 level as continuous group (*t* = 0.04, *P* = 0.969).

### Subgroup analysis

To evaluate the influences of CAD type, ethnicity, and follow-up duration on the role of the CXCL12 level in predicting CAD adverse outcomes, a subgroup analysis was conducted. According to the CAD type of study subjects, the included studies were divided into ACS subgroup and non-ACS subgroup; according to the ethnicity of study subjects, the included studies were divided into Asian subgroup and Caucasian subgroup; whereas based on different follow-up durations, the included studies were divided into short-term subgroup and long-term subgroup, respectively. The results of subgroup analysis stratified by CAD type, ethnicity, and follow-up duration are presented in Table 2. In the CXCL12 level as the category variable group, all subgroups stratified by CAD type, ethnicity, and follow-up duration (both in univariate and multivariate analyses) have significant correlations. However, in the CXCL12 level as the continuous variable group, the correlations of the ACS subgroup, Asian subgroup, and short-term subgroup were not statistically significant, though the correlations of non-ACS, Caucasian, and long-term subgroups were significant.

**Table 2:** Subgroup analysis stratified by CAD type, ethnicity, and follow-up duration.

**Subgroups**	**Category variable (*****n*** = **2,038)**				**Continuous variable (*****n*** = **921)**	
	**Univariate analysis**		**Multivariate analysis**		**Multivariate analysis**	
	**HR (95% CI)**	***I*** **^2^ (%)**	**HR (95% CI)**	**I^2^(%)**	**OR (95% CI)**	**I^2^ (%)**
ACS	9.72 (4.69–20.15)	70.4	2.47 (1.79–3.40)	0	1.36 (0.67–2.75)	85.3
Non-ACS	2.73 (1.65–4.54)	67.4	3.49 (1.66–7.33)	74.5	1.53 (1.23–1.92)	54.5
Asian	7.43 (1.70–32.49)	96.6	2.21 (1.71–2.85)	0	1.40 (0.91–2.14)	79.5
Caucasian	3.90 (2.73–5.57)	0	3.87 (2.48–6.04)	23.5	3.83 (1.44–10.19)	0
Short term^*^	9.36 (4.10–21.37)	78.5	2.72 (1.97–3.76)	45.3	1.16 (0.28–4.76)	89.1
Long term^*^	2.86 (1.62–5.04)	72.5	2.38 (1.76–3.22)	65	1.62 (1.37–1.93)	47

* *In the CXCL12 level as the category variable group, the short-term subgroup was defined as a follow-up period <24 months, while the long-term subgroup was defined as ≥24 months; in the CXCL12 level as the continuous variable group, the short-term subgroup was defined as a follow-up period <12 months, while long-term subgroup defined as ≥12 months*.

### Sensitivity analysis

**Figure 6:**
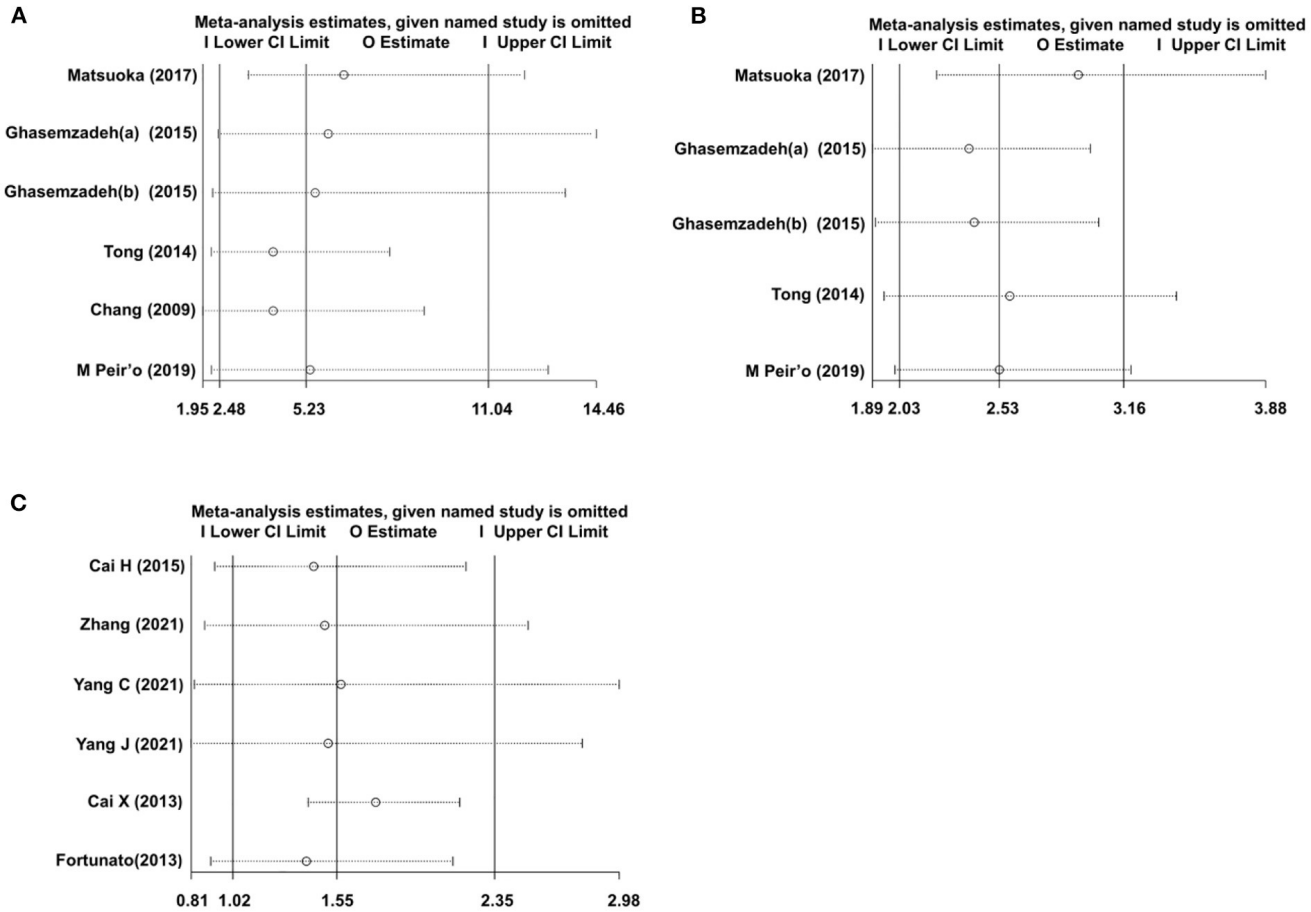
Sensitivity analysis evaluating the influence of any single study on the overall effects. **(A)** Univariate analysis data of the CXCL12 level as category variable group. **(B)** Multivariate analysis data of the CXCL12 level as category variable group; **(C)** Multivariate analysis data of the CXCL12 level as the continuous variable group.

To test the robustness of the pooled data of our meta-analysis, a sensitivity analysis was conducted. As a result, in the CXCL12 level as the category variable group, omitting any single study had no significant influence on the pooled HRs in both univariate analysis and multivariate analysis, indicating the robustness of pooled estimates (Figures 6A,B). However, in the CXCL12 level as the continuous variable group, each study except for the study by Cai X et al. had a significant influence on the overall effect (Figure 6C), suggesting the unstableness of the pooled estimate in this group.

## Discussion

CXCL12 (also referred to as SDF-1) is a member of the CXC chemokine family and plays a prominent role in hematopoiesis, angiogenesis, immunogenesis, stem cell mobilization, and tissue regeneration through its receptors CXCR4 and ACKR3 (7, 27). In the past two decades, the role of CXCL12 and CXCR4/ACKR3 systems in the pathogenesis of cardiovascular diseases emerged to be one of the research hotspots of this field (28). CXCL12 as a chemokine plays multifaceted roles in the pathogenesis of coronary atherosclerotic heart disease, both beneficial and detrimental roles of CXCL12 were reported (7, 29). A variety of studies reported that CXCL12 was cardioprotective after myocardial infarction, attenuated adverse ventricular remodeling, and preserved ventricular function after myocardial infarction (30, 31). Exogenous CXCL12 administration significantly alleviated myocardial ischemia/reperfusion injury (IRI) and improved post-ischemic myocardial functional recovery (32). In fact, considering the critical role of CXCL12 in promoting tissue repair and myocardial protection, a clinical trial aimed to improve cardiac function with the treatment of CXCL12 has been conducted. The STOP-HF randomized Phase II trial evaluated the safety and efficacy of a single treatment of plasmid CXCL12 delivered *via* endomyocardial injection to patients with ischemic heart failure and demonstrated the potential for attenuating left ventricular remodeling and improving ejection fraction (EF) in high-risk ischemic cardiomyopathy (33), further supporting the cardioprotective role of CXCL12.

However, other studies reported that the higher blood CXCL12 level correlated with the severity of coronary artery lesions and predicted adverse clinical outcomes in patients with stable CAD or acute coronary syndrome, though the underlying mechanism is unclear. Chang et al. (11) first reported that the higher serum CXCL12 level predicted 30-day major adverse clinical outcomes in patients with AMI. Thereafter, other studies also reported the correlation between higher blood CXCL12 levels and increased risk of future adverse clinical outcomes in patients with CAD (12–14). In contrast, negative or even opposite results were also reported (16). Thus, to evaluate the correlation between the blood CXCL12 level and the prognosis of CAD comprehensively and objectively, we conducted this meta-analysis.

By strict screening, 12 original studies with a total of 2,959 CAD subjects were entered into the final data combination. For different studies that assigned blood CXCL12 levels as different variable types, we first divided all the included studies into CXCL12 level as the category variable group and CXCL12 level as the continuous variable group and pooled the estimates, respectively. As a result, the pooled data indicated a significant association between higher CXCL12 levels and future adverse clinical events both in univariate analysis (pooled HR 5.23, 95% CI 2.48–11.04, *P* < 0.001) and multivariate analysis (pooled HR 2.53, 95% CI 2.03–3.16, *P* < 0.001) in CXCL12 level as category variable group. In the CXCL12 level as the continuous variable group, univariate data were available only in one study, so we only pooled the multivariate estimates, and the result also indicated that the higher CXCL12 level significantly predicted future adverse clinical events (pooled OR 1.55, 95% CI 1.02–2.35, *P* = 0.039). These results suggested that the blood CXCL12 level may be a valuable prognostic index for MACEs in patients with CAD.

Pathophysiologically, there are some differences between stable CAD and ACS (34), and different races may exert influences on the clinical characteristics and prognosis of CAD (35). In addition, the blood CXCL12 level may have different roles in predicting the short-term or long-term prognosis of CAD. So, the subgroup analysis stratified by CAD type, ethnicity, and follow-up duration was conducted to evaluate the influence of these three covariables on the overall effects. In the CXCL12 level as the category variable group, each subgroup (non-ACS or ACS, Caucasian or Asian, and short-term or long-term) showed a significant association between blood CXCL12 level and future MACEs. But in the CXCL12 level as the continuous variable group, the results were only significant in non-ACS, Caucasian, and long-term subgroups, suggesting the unstableness of the pooled OR in this group. In fact, sensitivity analysis also suggested that the pooled OR in the CXCL12 level as the continuous variable group was unstable, for several single studies, all had a significant influence on the overall pooled estimate (Figure 6C). In contrast, sensitivity analysis indicated that the pooled estimates were robust in the CXCL12 level as the category variable group, and no single study was indispensable for the significant overall HRs (Figures 6A,B).

Although all the included original studies measured CXCL12 level with ELISA, the detecting substrates were different. In the CXCL12 level as the continuous variable group, all the included studies detected CXCL12 level in serum, while in the CXCL12 level as the category group, only one in serum (the other five studies detected CXCL12 level in plasma) was detected. The composition of serum and plasma has a small difference, but the pooled estimates in both groups are all significant, indicating the consistency of the predicting role of CXCL12 level in serum and plasma.

Publication bias is a serious problem in the meta-analysis, which may affect the reliability and generalization of conclusions (36). In this meta-analysis, both Begg's and Egger's tests showed no significant publication bias in univariate and multivariable analyses of CXCL12 level as category variable group and multivariable analysis of CXCL12 level as the continuous variable group, indicating the authenticity and validity of the conclusions.

As for the mechanism underlying the association between higher blood CXCL12 levels and poor prognosis of CAD, it remains to be elucidated. But, existing clues indicated that higher blood CXCL12 level was associated with more severe coronary artery lesions (37), and CXCL12 promoted atherosclerosis to drive CAD progress (38), which may lead to a higher incidence of adverse cardiovascular events. This may partly account for the mechanism of the association between higher blood CXCL12 levels and poor prognosis of CAD.

Recently, Leberzammer et al. reported that CXCL12 augments platelet aggregation by activating its receptor CXCR4, while inhibition of CXCR4 attenuates platelet aggregation, and platelet-specific CXCL12 deficiency in mice limits arterial thrombosis, indicating the pro-thrombotic function of platelet-derived CXCL12 (39). In addition, an earlier study reported that platelet-derived CXCL12 can activate platelets thromboxane A2-dependently through its receptor CXCR4 (40). In contrast, higher expression of CXCL12 in platelets is associated with worse clinical outcomes in patients with CAD (41). In the CXCL12 level as the continuous variable group of this meta-analysis, all the original studies detected the CXCL12 level in serum, as much of serum CXCL12 may potentially be derived from circulating platelets activated during blood clotting, so the platelet-derived CXCL12 in serum may have exerted pro-thrombotic role to trigger adverse cardiovascular events. This further supports the role of higher blood CXCL12 levels in predicting the poor prognosis of CAD mechanistically.

CXCR4 and ACKR3 are the two receptors of CXCL12 known so far. CXCR4 is a G protein-coupled receptor (GPCR) and serves as an amplifier to increase CXCL12-associated signaling (42). ARKR3 does not couple with G protein; however, it has a much higher affinity for CXCL12 than CXCR4 and is initially considered a negative regulator of CXCL12 expression and function for the primary role of ACKR3 is to internalize and deliver CXCL12 for lysosomal degradation (43). ACKR3 has also been reported to be involved in signaling independent of G-protein (44). CXCR4 and ACKR3 perform both proatherogenic and athero-protective functions dependent on various cell types. Both CXCR4 and ACKR3 in macrophages are proatherogenic (45, 46), and CXCR4 in platelets was also reported to be proatherogenic (47). However, activation of CXCR4 or ACKR3 in vascular cells limits atherosclerosis progress (48, 49). We assumed that the proatherogenic role of CXCR4 in both macrophages and platelets and ACKR3 in macrophages is accountable for the association between higher blood CXCL12 levels and worse outcomes of CAD.

Atherosclerosis is an inflammatory disease (50), and CXCL12 was once considered a pro-inflammatory molecule (51), which may promote the progress of CAD and lead to a poor prognosis. However, later findings indicated that CXCL12 may have the opposite role in inflammation (52, 53). So, the actual mechanism underlying the correlation between higher blood CXCL12 levels and poor prognosis of CAD is complicated and warranted to be further explored.

To the best of our knowledge, this is the first meta-analysis assessing the association between blood CXCL12 levels and the prognosis of CAD. Inevitably, there are some limitations in our meta-analysis. First, as aforementioned, sensitivity analysis indicated the unstableness of pooled OR in the CXCL12 level as the continuous variable group, suggesting that using the CXCL12 level as the continuous variable to conduct multivariate logistic regression to assess the role of the CXCL12 level in predicting the prognosis of CAD maybe not a good method. Second, although we conducted subgroup analysis stratified by CAD clinical type, ethnicity, and follow-up duration, subgroup analysis stratified by different MACEs could not be conducted for lack of enough related data. Third, the sample size of a few included studies was small.

In summary, our meta-analysis illustrated that the higher blood CXCL12 level is associated with increased MACEs in patients with CAD, and the blood CXCL12 level may serve as an important prognostic index for CAD. Integrating blood CXCL12 levels into CAD risk assessment tools may provide more comprehensive messages for evaluating and managing patients with CAD, which are very beneficial for clinical workers. However, in considering the limitations of our meta-analysis, further large-scaled multicentered prospective studies are warranted to demonstrate the predicting role of the blood CXCL12 level in CAD prognosis, especially to elucidate its role in predicting specific MACEs.

## Data availability statement

The original contributions presented in the study are included in the article/supplementary material, further inquiries can be directed to the corresponding author.

## Author contributions

SZ: literature search, data collection, funds collection, and manuscript writing. YD and FF: data collection and interpretation. YG: study design, statistical analysis, and funds collection. All authors contributed to the article and approved the submitted version.

## Funding

This study was financially supported by the Zhejiang Medical and Health Science and Technology Project (Grant No. 2021KY234), the Hangzhou Medical and Health Science and Technology Project (Grant No. A20200804), and the Construction Fund of Key Medical Disciplines of Hangzhou (No. OO20200055).

## Conflict of interest

The authors declare that the research was conducted in the absence of any commercial or financial relationships that could be construed as a potential conflict of interest.

## Publisher's note

All claims expressed in this article are solely those of the authors and do not necessarily represent those of their affiliated organizations, or those of the publisher, the editors and the reviewers. Any product that may be evaluated in this article, or claim that may be made by its manufacturer, is not guaranteed or endorsed by the publisher.
